# Placental dysfunction drives fetal growth restriction: mechanisms and translational perspectives

**DOI:** 10.3389/fmolb.2026.1847608

**Published:** 2026-09-03

**Authors:** Ling He, Dan Hong, Huan Chen, Xiangyi Chen

**Affiliations:** 1 Department of Obstetrics, Maternal and Child Health Hospital of Hubei Province, Huazhong University of Science and Technology, Wuhan, China; 2 Laboratory Department of Wuhan Institute of Technology Hospital, Wuhan Institute of Technology, Wuhan, China

**Keywords:** angiogenic imbalance, fetal growth restriction, oxidative stress, placental dysfunction, precision obstetrics

## Abstract

Fetal growth restriction (FGR) is a major obstetric complication characterized by the inability of the fetus to reach its genetic growth potential, most commonly due to placental insufficiency. It contributes substantially to perinatal morbidity, stillbirth, and long-term cardiometabolic disease. Emerging evidence indicates that FGR arises not from a single defect but from a convergent, self-reinforcing network of placental dysfunction involving impaired trophoblast invasion, defective spiral artery remodeling, oxidative stress, angiogenic imbalance, immune dysregulation, nutrient transport failure, and epigenetic reprogramming. In early-onset FGR, hypoxia-driven HIF-1α signaling serves as an important integrating node linking angiogenic, inflammatory, and metabolic dysfunction, whereas additional HIF-1α-independent pathways appear to contribute more prominently in late-onset disease. Advances in single-cell and multi-omics technologies have refined the cellular and molecular landscape of the placenta, revealing complex intercellular interactions and regulatory circuits underlying disease heterogeneity. Clinically, diagnosis relies on integrated assessment of fetal biometry, Doppler velocimetry, and biomarkers such as the sFlt-1/PlGF ratio, while management remains largely surveillance-based with limited effective therapeutic options once placental dysfunction is established. Translational research is increasingly focused on precision approaches, including multi-omics biomarker panels, machine learning-based risk prediction, and pathway-targeted interventions. However, clinical implementation remains constrained by biological heterogeneity and lack of standardized validation. This review synthesizes current mechanistic insights and highlights emerging diagnostic and therapeutic strategies, emphasizing the need to shift from single-pathway models toward network-based and mechanism-stratified approaches. Advancing toward precision obstetrics will require integration of molecular profiling with clinical decision-making to improve outcomes for pregnancies affected by FGR.

## 1 Introduction

Fetal growth restriction (FGR) is defined as the failure of a fetus to achieve its genetically determined growth potential due to a pathological process, most commonly placental insufficiency (Gordijn et al., 2016). It affects approximately 10% of pregnancies globally and contributes to 30%–40% of all stillbirths, representing one of the most serious obstetric complications worldwide (Khalil et al., 2019; Lawn et al., 2016). Beyond the perinatal period, FGR exerts long-term effects, encompassing neurodevelopmental impairment, elevated risk of cardiovascular and metabolic disease in adulthood, and potential transgenerational epigenetic programming. These long-term sequelae are collectively explained by the developmental origins of health and disease (DOHaD) framework (Barker, 2004; D'Agostin et al., 2023).

While FGR can result from fetal chromosomal abnormalities, congenital infections, and maternal systemic disease, the predominant underlying mechanism is placental dysfunction arising from deficient remodeling of uterine spiral arteries and impaired uteroplacental perfusion (Aye et al., 2025; Figueras and Gratacós, 2014). Recent high-resolution molecular studies, including single-cell RNA sequencing (scRNA-seq) of the placental cellular landscape, have fundamentally reshaped our understanding of FGR pathogenesis. These studies have revealed that placental dysfunction does not arise from a single molecular defect but rather from a convergent pathological network encompassing oxidative stress, angiogenic imbalance, immune dysregulation, epigenetic reprogramming, and metabolic failure (Gaccioli et al., 2018; Vento-Tormo et al., 2018).

**FIGURE 1:**
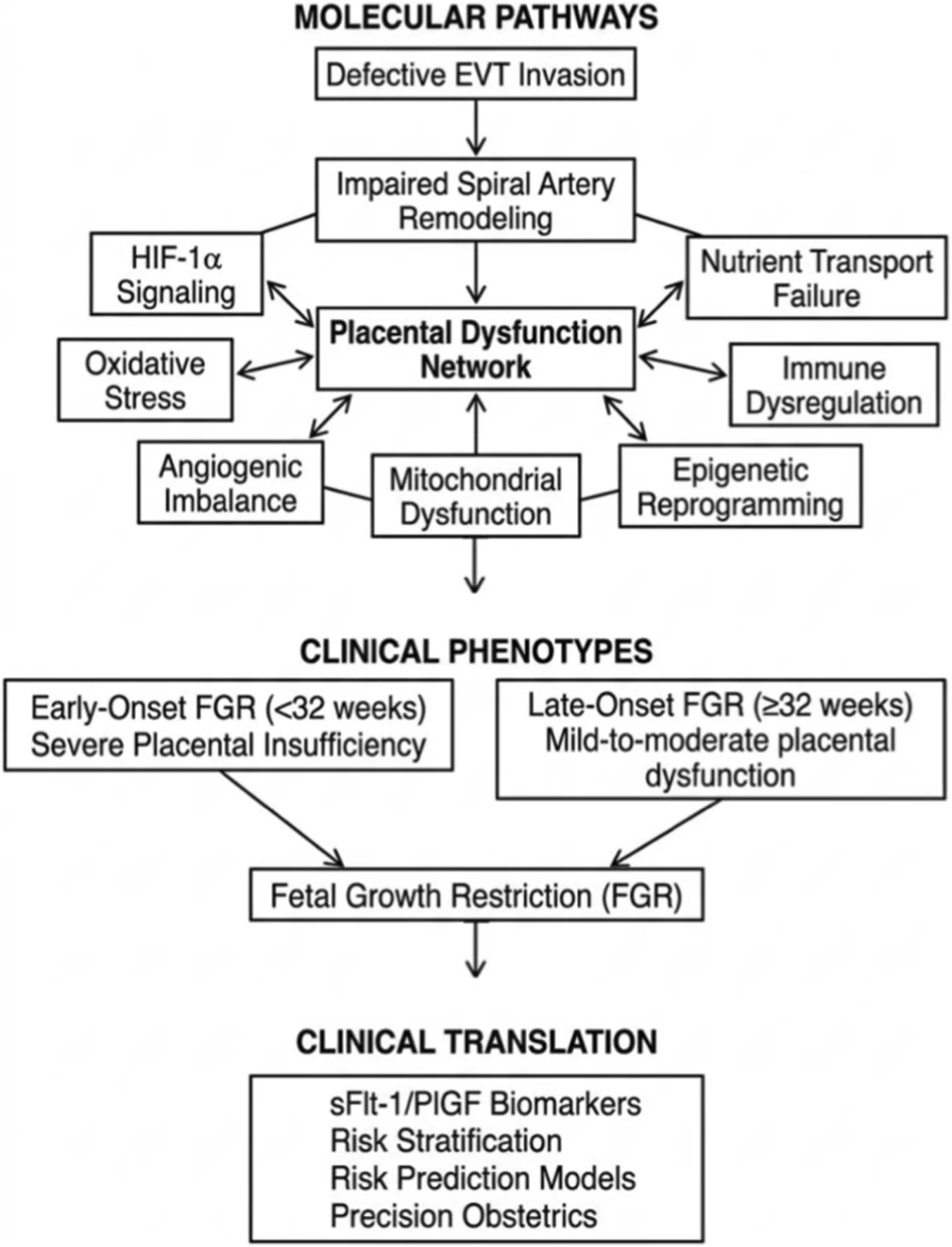
Graphical abstract illustrating the systems-level framework of FGR. Defective extravillous trophoblast (EVT) invasion and impaired spiral artery remodeling initiate a network of placental dysfunction involving HIF-1α signaling, oxidative stress, angiogenic imbalance, mitochondrial dysfunction, immune dysregulation, nutrient transport failure, and epigenetic reprogramming. These interconnected molecular pathways contribute to distinct clinical phenotypes, including early-onset and late-onset FGR, which ultimately converge on the shared outcome of impaired fetal growth. By linking molecular architecture to clinical heterogeneity, this framework provides a conceptual basis for risk prediction, biological stratification, and the eventual realization of precision obstetrics in FGR.

Clinically, accurate identification of growth-restricted fetuses, and their differentiation from constitutionally small but otherwise healthy fetuses, depends on the integration of fetal biometry, Doppler velocimetry, and validated biomarker panels (Lees et al., 2020). Management remains largely supportive, centered on optimal surveillance and delivery timing to balance the risks of intrauterine fetal compromise against iatrogenic prematurity (Figueras and Gratacos, 2014). Nevertheless, progress in translational research has yielded new diagnostic tools, including the sFlt-1/PlGF ratio, multi-omics-based biomarker signatures, and machine learning predictive models, while novel therapeutic targets are being explored in the context of precision obstetrics (Melamed et al., 2021; Mennickent et al., 2023). This review examines the molecular mechanisms underlying placental dysfunction in FGR, evaluates their translational implications for diagnosis and management, and identifies emerging research directions likely to reshape how this condition is approached clinically. The conceptual framework developed throughout this review is summarized in Figure 1, which illustrates the progression from placental molecular dysfunction to clinical phenotypes and translational applications in fetal growth restriction.

### 1.1 Literature search strategy and evidence appraisal

Literature searches were conducted using PubMed, Scopus, and Web of Science, incorporating studies available through the final revision of the manuscript. Search terms included combinations of “fetal growth restriction,” “placental dysfunction,” “trophoblast invasion,” “spiral artery remodeling,” “oxidative stress,” “angiogenic imbalance,” “sFlt-1,” “PlGF,” “HIF-1α,” “immune dysregulation,” “epigenetic regulation,” “nutrient transport,” “mitochondrial dysfunction,” “single-cell RNA sequencing,” “multi-omics,” and “precision obstetrics.” Additional relevant studies were identified through manual screening of reference lists from key articles and reviews.

Given the broad scope of this narrative review, no restrictions were applied based on study design. Greater emphasis was placed on evidence derived from human studies, including randomized controlled trials, prospective cohort studies, and systematic reviews or meta-analyses, particularly when discussing clinical diagnosis, prognosis, and management. Findings from human placental tissues were used to support mechanistic interpretation where appropriate. Experimental evidence from animal models and *in vitro* systems was incorporated primarily to provide mechanistic insight and is identified as such throughout the manuscript. When findings differed across study types, conclusions were guided by the highest-quality human evidence available. In areas where human data remain limited, experimental findings are presented as hypothesis-generating rather than definitive evidence.

## 2 Definition and classification

FGR is distinguished from small-for-gestational-age (SGA), which simply denotes a birth weight or estimated fetal weight (EFW) below the 10th percentile for gestational age, regardless of cause (Unterscheider et al., 2013). FGR specifically implies growth failure due to a pathological process, most often placental insufficiency, and is associated with elevated perinatal morbidity and mortality. We adopt the term FGR to maintain diagnostic consistency, subsuming relevant clinical data historically reported as intrauterine growth restriction (IUGR) throughout this review. The International Society of Ultrasound in Obstetrics and Gynecology (ISUOG) and the Delphi consensus criteria define FGR based on combinations of biometric thresholds, specifically an estimated fetal weight or abdominal circumference below the 3rd or 10th percentile for gestational age, in conjunction with abnormal Doppler parameters (Lees et al., 2020).

FGR is classified as early-onset, defined as delivery before 32 completed weeks of gestation, or late-onset, occurring at or after 32 weeks. These phenotypes differ substantially in their pathophysiology, severity, and clinical trajectory (Melamed et al., 2021). Early-onset FGR is typically associated with severe placental insufficiency, frequently co-occurs with preeclampsia, and is characterized by progressive Doppler deterioration including absent or reversed end-diastolic flow in the umbilical artery and abnormal ductus venosus (DV) waveforms (Lees et al., 2013). Late-onset FGR is more prevalent, often associated with milder placental dysfunction, and is recognized primarily by abnormal cerebroplacental ratio (CPR) reflecting brain-sparing redistribution (Vollgraff Heidweiller-Schreurs et al., 2018). A functional four-stage staging system based on Doppler parameters and biophysical profile scores guides clinical decision-making regarding delivery timing (Figueras and Gratacos, 2014).

## 3 Etiology

### 3.1 Placental and uterine factors

The most common cause of FGR is placental insufficiency secondary to impaired EVT invasion and defective spiral artery remodeling. During normal placentation between 8 and 18 weeks of gestation, EVT cells invade the decidua and myometrium, replacing the vascular smooth muscle and endothelium of the spiral arteries and converting them into wide-bore, low-resistance conduits (Lyall et al., 2013). In FGR, this transformation is incomplete, resulting in high-resistance, high-velocity maternal blood flow that causes repetitive ischemia-reperfusion injury to the placenta (Burton and Jauniaux, 2018). Histological examination of placental bed biopsies has confirmed significantly reduced myometrial spiral artery remodeling and fewer intramural trophoblasts in pregnancies complicated by preeclampsia and small-for-gestational-age births, with the degree of remodeling failure correlating with adverse perinatal outcomes (Khong et al., 1986).

Uterine structural anomalies, submucous fibroids, and antiphospholipid syndrome further impair uterine blood flow and trophoblast function. Uterine natural killer (uNK) cells and decidual macrophages play key regulatory roles in EVT invasion, and their dysfunction contributes to deficient remodeling (Crespo et al., 2020). Single-cell transcriptomic profiling of the maternal-fetal interface has further defined the molecular dialogue between EVTs and decidual immune cells, revealing dysregulated ligand-receptor interactions in FGR placentas (Vento-Tormo et al., 2018).

### 3.2 Maternal factors

Maternal conditions that impair uteroplacental perfusion or induce systemic inflammation are important FGR risk factors. Hypertensive disorders of pregnancy, including chronic hypertension and preeclampsia, directly reduce placental perfusion and amplify angiogenic imbalance through excess sFlt-1 secretion (Giardini et al., 2025). Autoimmune conditions such as systemic lupus erythematosus and antiphospholipid syndrome promote thrombotic microangiopathy of the decidual vasculature. Maternal smoking is independently associated with FGR via increased carboxyhemoglobin, vasoconstriction, and direct trophoblast toxicity, reducing birth weight by 150–300 g on average (Figueras and Gratacós, 2014; Figueras and Gratacos, 2014). Severe malnutrition, particularly protein deficiency and micronutrient insufficiency, including deficits in iron, folate, and zinc, impairs placental growth factor synthesis and nutrient transport capacity (Jansson and Powell, 2013).

### 3.3 Fetal and environmental factors

Chromosomal abnormalities, most commonly trisomies 13, 18, and 21 as well as confined placental mosaicism, account for 5%–10% of FGR cases, particularly early-onset forms with severe biometric discordance (Figueras and Gratacós, 2014). Congenital infections, including cytomegalovirus (CMV), toxoplasma, and rubella, impair trophoblast function and fetal cell replication. Environmental factors including altitude-induced hypoxia, endocrine-disrupting chemicals and exposure to fine particulate matter are increasingly recognized FGR contributors. Recent experimental work has shown that carbon black nanoparticle exposure disrupts placental mitochondrial autophagy, triggering ferroptosis and fetal growth restriction in murine models (Zhang et al., 2025).

## 4 Molecular network mechanisms underlying placental dysfunction

**FIGURE 2:**
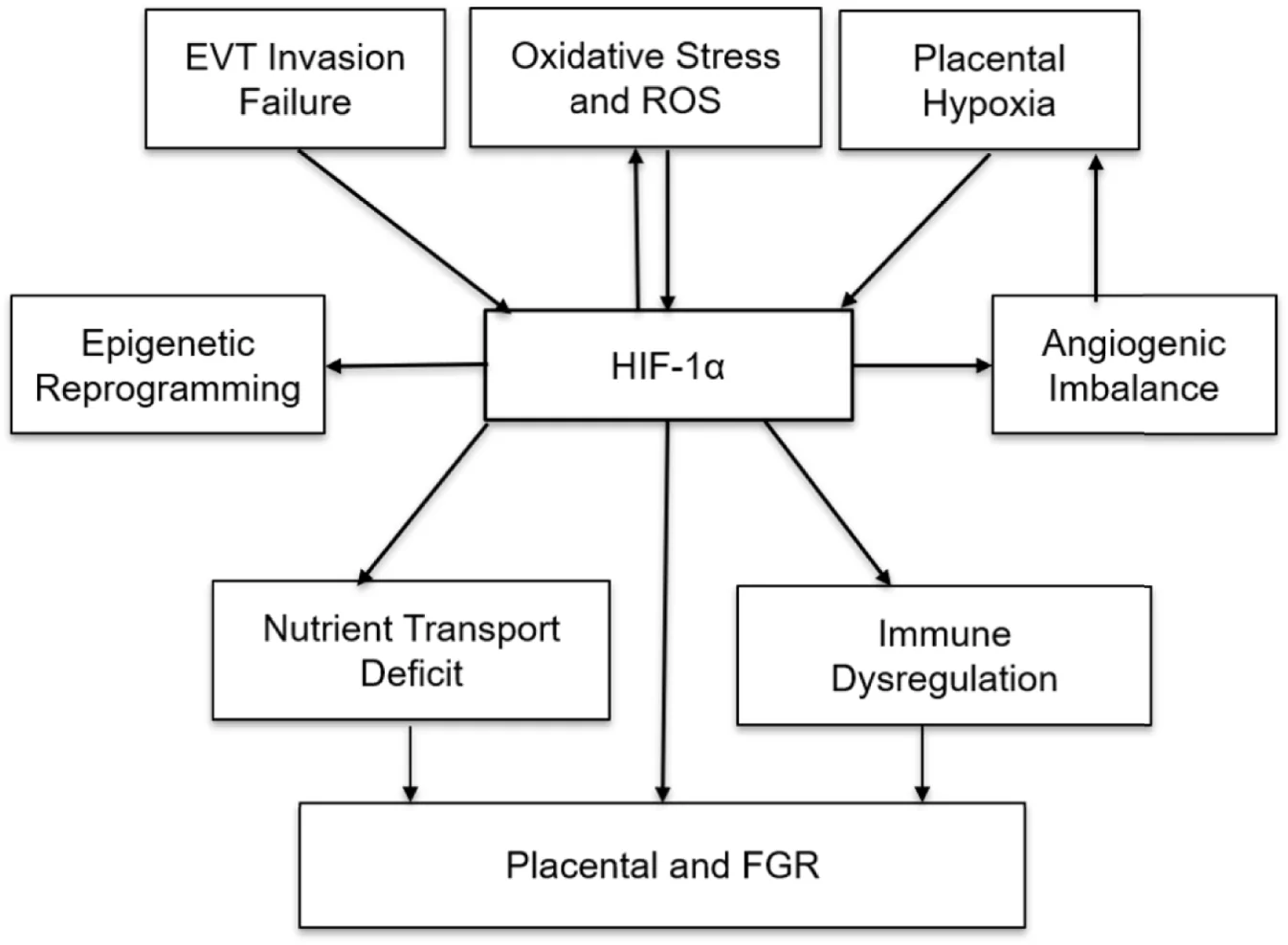
Network-based model of placental dysfunction underlying FGR. FGR reflects a convergent and self-reinforcing network of placental pathological mechanisms rather than a single dominant pathway. Defective EVT invasion disrupts uteroplacental perfusion and promotes chronic hypoxia, leading to stabilization of HIF-1α, an important regulatory node within the placental stress response network. Acting as a transcriptional hub, HIF-1α coordinates multiple downstream programs, including oxidative stress and ROS generation, angiogenic imbalance, immune dysregulation, impaired nutrient transport, and epigenetic reprogramming. These pathways are dynamically interconnected, with feedback interactions between hypoxia and oxidative stress and further cross-talk across angiogenic and immune axes.

Placental dysfunction in FGR reflects a convergent, self-reinforcing molecular network rather than a collection of parallel pathological processes, though the relative contribution of each axis likely varies across clinical subtypes and gestational windows. HIF-1α stabilization secondary to deficient spiral artery remodeling can drive excess sFlt-1 production, suppress PlGF synthesis, amplify mitochondrial ROS generation, and activate NF-κB, thereby linking angiogenic, oxidative, and inflammatory pathways. This role appears particularly important in early-onset FGR, where severe uteroplacental hypoxia is a dominant pathological feature. However, HIF-1α should be viewed as an important context-dependent node rather than a universal master regulator. Additional pathways involving inflammatory signaling, mitochondrial dysfunction, nutrient sensing, and epigenetic regulation may contribute independently to placental dysfunction, particularly in late-onset FGR where placental hypoxia is often less pronounced. This network topology carries important therapeutic implications, with disruption of any single node propagating dysfunction across the others and thereby likely explaining the limited efficacy of single-agent interventions in established FGR. The integrated pathological cascade is depicted in (Figure 2). Understanding these interconnections is critical for identifying molecular nodes amenable to therapeutic intervention.

It is important to recognize that placental studies of FGR are shaped by substantial biological and clinical heterogeneity before considering individual molecular pathways. Maternal characteristics, including hypertensive disorders, diabetes, obesity, and other comorbidities, influence placental vascular, metabolic, and inflammatory adaptations and may contribute to differences among study cohorts (Burton and Jauniaux, 2018). Increasing evidence also indicates that placental responses to hypoxia and nutrient stress differ between male and female fetuses, although sex-specific analyses remain underreported in many mechanistic investigations (Chatterjee et al., 2022). Interpretation is further complicated by gestational age, as placental structure and transcriptional programs evolve throughout pregnancy, making direct comparisons between early- and late-onset FGR inherently challenging (Burton et al., 2014). Even within the same placenta, regional variation in cellular composition and oxygen exposure can influence molecular measurements, highlighting the importance of standardized sampling strategies. Recognition of these sources of heterogeneity is essential for interpreting mechanistic findings, evaluating biomarkers, and improving the reproducibility of future translational studies.

### 4.1 Impaired trophoblast invasion and spiral artery remodeling

EVT invasion is a tightly regulated, multi-step process governed by growth factors, proteases, adhesion molecules, and transcription factors. The full network of regulatory interactions is still being worked out (Lyall et al., 2013). The HIF-1α/KDM3A/MMP-12 regulatory circuit has been implicated in regulating trophoblast plasticity under low-oxygen conditions during early placentation. Trophoblast organoid studies have corroborated a role for HIF-1α-dependent transcriptional programs in trophoblast differentiation and vascular remodeling capacity (Haider et al., 2018). MMP-2 and MMP-9 facilitate EVT migration through extracellular matrix degradation, while their endogenous inhibitors, the TIMPs, are dysregulated in FGR placentas. IGF-2 functions as a critical paracrine regulator of EVT invasion. Placental-specific IGF-2 knockout mice develop severe FGR with markedly reduced labyrinthine surface area (Constância et al., 2002).

Single-cell RNA sequencing of FGR placentas has revealed reduced proportions of EVT cell subtypes and significantly decreased expression of fibronectin 1, an ECM adhesion molecule critical for EVT anchoring and migration (Hua et al., 2024). Complementary single-cell transcriptomic analyses have defined the full spectrum of trophoblast subtype diversity in the human placenta, identifying distinct cytotrophoblast progenitor, syncytiotrophoblast, and EVT subpopulations with characteristic differentiation trajectories. These reference maps provide a cellular framework in which FGR-associated transcriptional defects, such as the FN1 reduction observed in FGR EVTs, can be precisely localized and interpreted (Hua et al., 2024; Suryawanshi et al., 2018). MicroRNA-155-5p (miR-155-5p), acting via suppression of FOXO3, and other placenta-enriched miRNAs from the chromosome 19 miRNA cluster function as post-transcriptional regulators of EVT invasion. Their dysregulation in FGR suggests that the trophoblast invasion defect is not simply a failure of upstream growth factor signaling but is compounded by disruption of the miRNA networks that fine-tune cell behavior at the post-transcriptional level (Hayder et al., 2022; Hou et al., 2021).

Angiopoietin-2, expressed by both trophoblasts and decidual cells, promotes EVT migration and invasion through Tie2/JNK/c-Jun signaling and upregulation of MMP-2 and MMP-9. Perturbation of the Angiopoietin-2/Angiopoietin-1 ratio in the FGR decidua disrupts the pro-invasive microenvironment and impairs spiral artery remodeling (Hou et al., 2021). The PI3K/AKT/FOSL1 axis and ASCL2 transcription factor are conserved regulators of the invasive trophoblast lineage across hemochorial species, providing potential therapeutic targets (Varberg and Soares, 2021).

### 4.2 Oxidative stress and mitochondrial dysfunction

Oxidative stress is a central and early feature of FGR-associated placental dysfunction, arising when reactive oxygen species (ROS) production exceeds the antioxidant defense capacity of the trophoblast (Brooker et al., 2024). Ischemia-reperfusion cycles resulting from incomplete spiral artery remodeling generate bursts of mitochondrial reactive oxygen species that activate NF-κB and HIF-1α, amplify inflammatory signaling, and directly damage syncytiotrophoblast membranes. In selective FGR placentas, a recent study employing transmission electron microscopy and targeted mitochondrial sequencing documented pronounced cytotrophoblast mitochondrial damage, augmented mitochondrial copy number in syncytiotrophoblast as a compensatory response, elevated oxidative stress markers, reduced ATP generation, and altered expression of mitochondrial long non-coding RNAs, collectively confirming energetic failure as a core FGR mechanism (Hu et al., 2024).

The Nrf2/Keap1/ARE antioxidant pathway constitutes a critical protective response to oxidative stress in trophoblasts. In FGR trophoblasts, Nrf2-mediated ferroptosis suppression, which operates through GPX4 and the SLC7A11 cystine transporter, is functionally impaired, and pharmacological restoration of Nrf2 activity attenuates trophoblast ferroptotic cell death, though most of this evidence comes from cell-line or murine studies rather than human tissue (Han et al., 2024; Kweider et al., 2017). Nrf2 deletion in mice impairs placental labyrinthine development and reduces labyrinthine volume, confirming its importance for placental structural integrity (Kweider et al., 2017). Ascorbate, melatonin, and N-acetylcysteine have been proposed as strategies to mitigate placental oxidative stress in FGR, though robust evidence of efficacy in FGR-specific experimental models remains limited (Han et al., 2024).

Mitochondrial fission and fusion dynamics are increasingly recognized as important regulators of trophoblast function. Accumulating evidence indicates that dysregulation of mitochondrial fusion and fission dynamics, impaired mitophagy, and activation of the mitochondrial unfolded protein response collectively contribute to placental dysfunction in FGR. Antioxidant supplementation with coenzyme Q10 and melatonin can partially restore mitochondrial function in experimental models (Wu et al., 2024). Ferroptosis is an iron-dependent form of regulated cell death driven by lipid peroxidation and has recently been implicated in trophoblast dysfunction in FGR, particularly under environmental exposure conditions. Arsenic exposure induces ferroptosis in HTR-8/SVneo cells through suppression of the Nrf2/GPX4 pathway and disruption of iron homeostasis, suggesting a potential mechanistic link between environmental toxins and FGR (Dong et al., 2024). Experimental studies have also shown that carbon black nanoparticle induced FGR in mice involves an autophagy and ferroptosis regulatory axis. In this model, lysosomal dysfunction impairs mitophagy, leading to accumulation of dysfunctional mitochondria and triggering lethal lipid peroxidation in trophoblasts (Zhang et al., 2019).

Most of the evidence implicating oxidative stress and ferroptosis in FGR has been generated from experimental models, including cell culture systems and animal studies. Human data are comparatively limited and are derived mainly from early-onset FGR or mixed cohorts. As a result, the relevance of these findings to late-onset FGR is still uncertain and requires further investigation.

### 4.3 Angiogenic imbalance of the sFlt-1/PlGF axis

Among the molecular mechanisms implicated in FGR, angiogenic imbalance is arguably the most clinically actionable at present, because circulating sFlt-1 and PlGF can be measured serially in maternal blood. Whether this translational advantage will eventually extend to the other pathological axes remains to be seen. Under normal conditions, placental growth factor (PlGF) and vascular endothelial growth factor (VEGF) promote uteroplacental angiogenesis, while soluble fms-like tyrosine kinase-1 (sFlt-1) acts as an endogenous antagonist that sequesters these factors, maintaining homeostatic balance (Verlohren et al., 2022). In placental insufficiency, sFlt-1 production rises substantially, driven by hypoxia-induced HIF-1α and compounded by reduced PlGF output from dysfunctional trophoblasts, generating a state of unopposed anti-angiogenic dominance (Giardini et al., 2025).

The sFlt-1/PlGF ratio has emerged as the most clinically validated biochemical marker of placental dysfunction. A landmark systematic review and meta-analysis demonstrated that a sFlt-1/PlGF ratio above 33 predicts FGR with pooled sensitivity of 0.63 and specificity of 0.84, yielding an area under the curve of 0.84, while a ratio of 85 or above yields sensitivity of 0.79 and specificity of 0.69 (Chen et al., 2022). Comprehensive analyses of clinical studies have demonstrated that the sFlt-1/PlGF ratio provides valuable prognostic information in both singleton and twin pregnancies, particularly for predicting disease progression within one to 4 weeks, guiding delivery decisions, and monitoring established FGR (Stepan et al., 2023). A recent retrospective cohort confirmed that elevated sFlt-1/PlGF (>85) in FGR pregnancies is independently associated with intrauterine fetal death, aberrant Doppler, and higher rates of obstetric complications compared with FGR with normal ratios (Arnouts et al., 2025).

The distinct contributions of sFlt-1 and PlGF carry different diagnostic and biological implications. Elevated sFlt-1 is the dominant driver of anti-angiogenic dominance in preeclampsia. Reduced PlGF, by contrast, reflects impaired trophoblast mass and function and correlates more closely with the severity of FGR itself (Gaccioli et al., 2023). Clinical studies in cohorts of preterm FGR pregnancies further indicate that low PlGF levels represent the primary biomarker of fetal compromise. This reduction is attributed to decreased trophoblast production, whereas elevated sFlt-1 mainly reflects increased binding and sequestration of circulating angiogenic factors (Gaccioli et al., 2023). Soluble endoglin is a co-receptor of transforming growth factor beta 1 that interferes with endothelial nitric oxide synthesis. Circulating soluble endoglin levels are elevated in FGR and contribute to placental vascular dysfunction through additional anti-angiogenic mechanisms (Verlohren et al., 2022).

### 4.4 Immune dysregulation at the maternal-fetal interface

At the maternal-fetal interface, immune tolerance and trophoblast invasion are inseparably coordinated by decidual natural killer (dNK) cells, regulatory T cells (Tregs), and decidual macrophages (dMs). dNK cells, which constitute 70% of decidual lymphocytes in early pregnancy, are critical for spiral artery remodeling and EVT guidance. When dNK cell numbers are reduced or their function is impaired, vascular remodeling is disrupted and trophoblast invasion is excessively restricted. The mechanisms governing dNK cell recruitment and activation in early pregnancy are incompletely understood, which makes this pathway difficult to target therapeutically (Crespo et al., 2020).

Decidual macrophages adopt a spectrum of phenotypes with functionally opposing roles. Current evidence, synthesized in a recent review encompassing both original data and published literature, indicates that a shift toward pro-inflammatory M1 polarization in FGR decidua, characterized by elevated TNF-α, IL-6, and CXCL16 secretion, is associated with impaired trophoblast invasion and reduced uteroplacental perfusion (Bezemer et al., 2024). Complementary single-cell transcriptomic data distinguished two functionally distinct decidual macrophage subsets in early human pregnancy, with the barrier-associated subset showing characteristics consistent with expansion in placental dysfunction states (Vondra et al., 2023). Hofbauer cells, the fetal-derived resident macrophages of the placental villous stroma, also modulate villous angiogenesis and are functionally altered in FGR, showing impaired capacity to support vasculogenesis (Reyes and Golos, 2018).

CXCL16 has been proposed as a mechanistic link between immune dysregulation and trophoblast dysfunction. Elevated CXCL16 in FGR decidua, produced primarily by pro-inflammatory macrophages, appears to inhibit EVT migration through its receptor CXCR6, though direct causality in human FGR has not yet been established (Bezemer et al., 2024). The NF-κB signaling cascade downstream of pro-inflammatory cytokines amplifies reactive oxygen species production and sFlt-1 secretion, creating a biochemical bridge between immune dysregulation and the oxidative and angiogenic axes. Notably, hypoxia itself promotes NF-κB activation through HIF-1α-independent mechanisms, which means that even partial restoration of placental perfusion may be insufficient to resolve the inflammatory state once it is established. This self-sustaining quality of the immune-inflammatory response is, in our view, one of the more underappreciated aspects of FGR pathophysiology (Gaccioli et al., 2018). Tregs constitute a critical immunosuppressive population at the maternal-fetal interface; their reduction in peripheral blood and decidua has been consistently documented in FGR, and experimental depletion of Tregs in murine pregnancy models recapitulates features of spiral artery remodeling failure (Inada et al., 2013). Furthermore, an imbalanced Treg/Th17 ratio, with relative predominance of pro-inflammatory interleukin-17-producing cells, has been identified in FGR decidua and is associated with the degree of placental inflammatory infiltrate and trophoblast invasion deficiency (Lee et al., 2012). Immune dysregulation in FGR does not appear to be simply a matter of excess inflammation. The data point instead to a structurally self-reinforcing imbalance in which each component, the shift toward M1 macrophages, the reduction in Tregs, and the elevation of pro-inflammatory cytokines, tends to perpetuate the others.

Current evidence for immune dysregulation in FGR is derived largely from early-onset FGR and mixed clinical cohorts. Data from late-onset FGR remain limited, making it difficult to determine whether alterations in dNK cells, Tregs, and macrophage polarization are shared across all forms of the disease. It is also important to note that mechanistic depletion studies have been performed almost exclusively in animal models.

### 4.5 Nutrient transport defects

The placenta is the primary conduit for fetal nutrient supply, and impaired nutrient transport is a direct mediator of FGR. Glucose uptake across the syncytiotrophoblast depends principally on the facilitative transporter GLUT-1, encoded by SLC2A1, whose protein expression is significantly reduced across both microvillous and basal membranes in FGR placentas, thereby limiting fetal glucose availability (Jansson et al., 1993). System A amino acid transporters, comprising the isoforms SNAT1, SNAT2, and SNAT4, encoded by SLC38A1, SLC38A2, and SLC38A4 respectively, mediate uptake of neutral amino acids including alanine, serine, and glutamine; their collective activity is reduced in FGR in proportion to disease severity, contributing directly to fetal amino acid deprivation (Jansson and Powell, 2013; Roos et al., 2007). Reduced expression of fatty acid transport proteins and fatty acid binding protein limits placental transfer of long-chain polyunsaturated fatty acids, particularly docosahexaenoic acid, with adverse consequences for fetal brain and retinal development (Roos et al., 2007; Larqué et al., 2011).

The mechanistic target of rapamycin complex 1 (mTORC1) functions as a master intracellular nutrient sensor, integrating signals from amino acid availability, oxygen tension, and growth factors to regulate placental transport capacity. In human FGR placentas, mTORC1 signaling is downregulated in proportion to restriction severity, with a corresponding decline in leucine transport capacity, establishing this axis as a central mediator of fetal amino acid deprivation (Roos et al., 2007). Reduced mTORC1 signaling in the FGR placenta downregulates System A transporter expression and activity, creating a self-reinforcing restriction of fetal nutrient supply that worsens as placental function declines. It is worth noting that the relative importance of glucose versus amino acid deprivation in determining fetal growth trajectory is not fully resolved, and may differ between early- and late-onset FGR (Jansson and Powell, 2013). In experimental models of uteroplacental insufficiency, targeted mTORC1 activation partially restores amino acid transporter expression and improves fetal weight, though translation to clinical intervention remains premature (Kavitha et al., 2014). Complementary evidence from protein-restricted FGR mouse models demonstrates parallel downregulation of placental mTOR signaling, insulin/IGF-I signaling, and System A transporters, confirming that this axis is responsive to maternal nutritional status and represents a potential point of intervention (Kavitha et al., 2014).

### 4.6 Epigenetic regulation and transgenerational programming

Epigenetic mechanisms, including DNA methylation, histone modification, and non-coding RNA regulation, are now understood to be active mediators of placental dysfunction in FGR, not merely secondary consequences of it. Their particular relevance lies in the capacity to alter gene expression without changing the underlying DNA sequence, thereby sustaining transcriptional abnormalities well beyond the initial hypoxic insult and potentially transmitting them to subsequent generations (Salmeri et al., 2022). The IGF2/H19 imprinted domain on chromosome 11p15 is critical for placental and fetal growth. Hypomethylation of the differential methylation region (DMR) disrupts normal IGF2 expression and is consistently observed in FGR placentas, suggesting that epigenetic destabilization of this locus is a recurring feature of placental insufficiency (Salmeri et al., 2022). Genome-wide DNA methylation profiling of human placentas from pregnancies complicated by preeclampsia and fetal growth restriction has identified promoter hypomethylation across multiple gene loci, indicating that epigenetic dysregulation is a shared feature of placental insufficiency syndromes with potential relevance to trophoblast differentiation and vascular signaling (Salmeri et al., 2022).

Non-coding RNAs play particularly important roles. MiR-210, stabilized by HIF-1α, suppresses key mitochondrial electron transport chain components, including ISCU1/2 and NDUFA4, and promotes glycolytic metabolism in hypoxic trophoblasts, contributing to energetic dysfunction (Kochhar et al., 2022). Recent studies characterized broader miRNA dysregulation networks in FGR placentas, identifying panels of differentially expressed miRNAs targeting cell cycle, apoptosis, and angiogenic pathways that hold potential as circulating biomarkers (Kochhar et al., 2022; Hromadnikova et al., 2019). LncRNA H19, which is transcribed from the opposite strand of the IGF2 locus, regulates trophoblast cell cycle progression and invasion (Zuckerwise et al., 2016). LncRNA DANCR similarly modulates the EVT phenotype, and both are dysregulated in FGR (Gu et al., 2013).

Emerging evidence raises the possibility that placental epigenetic programming in FGR may transmit disease risk to the next-generation through germline epigenetic marks. The data supporting this remain preliminary, and disentangling true epigenetic transmission from the effects of shared genetic variants is methodologically difficult in human cohort studies. This remains an active and somewhat contested area (Salmeri et al., 2022). This provides a molecular basis for the observation that FGR offspring show elevated rates of cardiometabolic disease and potentially transmit increased placentation risk to their own pregnancies, creating heritable cycles of poor fetal growth.

Evidence supporting epigenetic mechanisms in FGR is derived from both human and experimental studies, although the relative contribution of individual pathways remains incompletely defined. Findings related to IGF2/H19 methylation are based largely on human placental tissues, whereas mechanistic studies of miR-210 have relied predominantly on cell culture and animal models. Evidence for transgenerational epigenetic inheritance in humans remains limited.

### 4.7 An integrated pathophysiological model of FGR

The mechanistic axes described above are not independent. They form an integrated, self-amplifying network in which early perturbation of one pathway tends to propagate dysfunction across the others. HIF-1α occupies a central position within this network and serves as an important mediator of placental responses to hypoxic stress. Evidence supporting this role is strongest in early-onset FGR, where severe uteroplacental hypoxia is a prominent feature. However, HIF-1α should not be interpreted as a universal driver of placental dysfunction. Additional pathways involving inflammatory signaling, mitochondrial dysfunction, nutrient sensing, and epigenetic regulation may contribute independently to disease pathogenesis, particularly in late-onset FGR. These observations support a model in which FGR arises from multiple interacting and context-dependent pathways rather than a single dominant regulatory axis. This systems-level perspective, increasingly supported by multi-omics data from human placental studies, represents a meaningful shift from earlier single-pathway models, though the precise topology and relative weighting of these interactions continue to be delineated (Rahnavard et al., 2024). Available evidence suggests the following framework. Deficient EVT invasion and failed spiral artery remodeling generates the primary lesion of sustained uteroplacental hypoxia. Hypoxia stabilizes HIF-1α, which is proposed to simultaneously drive excess sFlt-1 production, suppress PlGF synthesis, induce mitochondrial reactive oxygen species generation, and activate NF-κB, thereby coupling the angiogenic, oxidative, and inflammatory axes within converging transcriptional responses (Burton and Jauniaux, 2018).

Activation of NF-κB signaling downstream is proposed to promote pro-inflammatory M1 polarization of decidual macrophages. This shift in immune balance may impair Treg function and further restrict trophoblast invasion, thereby reinforcing the underlying defect in placentation (Bezemer et al., 2024). Oxidative stress is also proposed to impair mTORC1 signaling in the syncytiotrophoblast, contributing to reductions in GLUT-1 and System A transporter expression that limit fetal glucose and amino acid delivery (Jansson and Powell, 2013). HIF-1α further stabilizes miR-210, which suppresses mitochondrial electron transport chain function and may reinforce energetic failure in hypoxic trophoblasts (Salmeri et al., 2022). Epigenetic modifications, including IGF2/H19 locus hypomethylation and HIF-1α-associated chromatin remodeling, may contribute to persistent transcriptional changes that sustain trophoblast dysfunction and nutrient transport deficiency beyond the initial hypoxic insult, though the durability of these changes in human FGR requires further investigation (Constância et al., 2002).

This network-level framework has important translational implications. The limited success of single-agent therapies in established FGR may reflect the simultaneous activation of multiple pathogenic pathways rather than dysfunction of a single molecular target (Sharp et al., 2018). Future advances will likely depend on aligning therapeutic strategies with the underlying biological heterogeneity of the disease. The major molecular pathways involved in placental dysfunction and their translational implications are summarized in Table 1.

**TABLE 1:** Major molecular pathways in placental dysfunction and their translational implications.

Pathway	Key molecules	Placental effect	Therapeutic target	Translational readiness	Key references
Trophoblast invasion	HIF-1α, MMP-2/9, IGF-2, FN1, ASCL2	Deficient spiral artery remodeling, reduced EVT migration	PI3K/AKT/FOSL1, MMP activators	Research	Lyall et al. (2013), Haider et al. (2018), Hua et al. (2024), Varberg and Soares (2021)
Oxidative stress	ROS, Nrf2/GPX4, NF-κB, mtDNA	Syncytiotrophoblast damage, ferroptosis, energetic failure	Nrf2 activators, melatonin, NAC, CoQ10	Preclinical	Brooker et al. (2024), Wu et al. (2024)
Angiogenic imbalance	sFlt-1, PlGF, VEGF, sEng	Endothelial dysfunction, vascular insufficiency	Anti-sFlt-1 RNAi, recombinant VEGF (preclinical)	Clinical biomarker	Verlohren et al. (2022), Chen et al. (2022), Gaccioli et al. (2023)
Immune dysregulation	dNK cells, Tregs, Th17, decidual macrophages, CXCL16	Impaired maternal-fetal tolerance, trophoblast invasion restriction	Low-dose IL-2 (Treg expansion), M2 macrophage polarization	Preclinical	Crespo et al. (2020), Bezemer et al. (2024), Vondra et al. (2023)
Mitochondrial dysfunction	mtROS, BNIP3, miR-210, DRP1/MFN2	Reduced ATP, impaired trophoblast bioenergetics	CoQ10, melatonin, mitophagy inducers	Preclinical	Wu et al. (2024)
Nutrient transport failure	GLUT-1, SNAT1/2/4, FATPs, mTORC1, IGF-2	Reduced fetal glucose, amino acid and lipid delivery	mTOR pathway modulation (experimental models), nutritional supplementation	Preclinical	Jansson and Powell (2013), Jansson et al. (1993)
Epigenetic reprogramming	IGF2/H19 DMR, miR-210, lncRNA H19, DANCR	Transgenerational programming, heritable cardiometabolic risk	DNMT modulators, miRNA inhibitors (research stage)	Research	Constância et al. (2002), Salmeri et al. (2022)

Translating mechanistic insights into clinical applications remains a major challenge in FGR research. Among the pathways discussed above, angiogenic imbalance has had the clearest translational impact, leading to clinically validated biomarkers that are increasingly incorporated into risk assessment and disease monitoring. In contrast, evidence linking oxidative stress, immune dysregulation, mitochondrial dysfunction, and epigenetic alterations to FGR derives largely from experimental and preclinical studies. Although these pathways have advanced our understanding of disease pathogenesis, their value as diagnostic or therapeutic targets remains uncertain. Differences in translational maturity should be considered when interpreting the current literature. Not all mechanisms that contribute to placental dysfunction have yielded clinically actionable tools, and in many cases the biological rationale remains ahead of clinical validation.

## 5 Clinical applications in FGR diagnosis and monitoring

The diagnosis of FGR relies on a multimodal framework integrating fetal biometry, Doppler velocimetry, and placental biomarkers. No single parameter provides adequate sensitivity and specificity across the full gestational spectrum, and this limitation is not simply a matter of technological shortcoming but reflects the biological heterogeneity of the condition itself. Early-onset FGR is characterized by prominent Doppler abnormalities and angiogenic imbalance, whereas late-onset FGR, the more prevalent phenotype, often presents with normal umbilical artery flow and requires reliance on cerebroplacental ratio and subtle biometric deceleration for timely detection.

### 5.1 Fetal biometry and growth standards

Fetal biometry is the foundation of FGR diagnosis. Abdominal circumference is the most sensitive single parameter, reflecting hepatic glycogen stores and subcutaneous fat depletion (Lees et al., 2020). Estimated fetal weight (EFW) derived from the Hadlock formula using head circumference, abdominal circumference, and femur length provides comprehensive assessment. The choice of growth reference chart carries significant diagnostic implications, as the INTERGROWTH-21st prescriptive standard and the WHO fetal growth charts yield higher FGR detection rates than population-based norms by identifying constitutionally appropriate fetuses who would otherwise be missed using local percentile references (Papageorghiou et al., 2018). Serial biometry assessing interval growth velocity is essential. A fetus that is not small in absolute size but demonstrates sustained deceleration across centile lines may still represent clinically significant FGR and warrants further investigation.

### 5.2 Doppler velocimetry

Despite its established role in FGR surveillance, Doppler velocimetry carries important clinical limitations that affect its application across the gestational spectrum. In early-onset FGR, differences in recommendations between major guidelines regarding the additive value of middle cerebral artery and ductus venosus Doppler have created inconsistency in clinical practice, and a evidence-based review concluded that the combined finding of an estimated fetal weight below the 10th centile with abnormal umbilical artery Doppler remains the most widely accepted indicator of placental disease (Lees et al., 2022). In late-onset FGR, the challenge is more pronounced, as umbilical artery pulsatility index frequently remains within the normal range and no individual Doppler parameter reliably predicts sudden fetal deterioration or intrapartum compromise in this phenotype. Cerebroplacental ratio below the fifth centile improves detection in this group but carries only moderate predictive accuracy when used alone, and its combination with estimated fetal weight and clinical risk factors is increasingly recommended to guide individualized management (Morales-Roselló et al., 2024). Across both phenotypes, Doppler findings should therefore be interpreted alongside biometry, biomarkers, and computerized cardiotocography rather than as standalone triggers for intervention.

### 5.3 Biomarkers from angiogenic factors to multi-omics

For placental dysfunction-related FGR, the sFlt-1/PlGF ratio remains the most robustly validated biochemical marker. A meta-analysis of eight studies reported a pooled AUC of 0.84 for FGR prediction at a threshold of 33 (Chen et al., 2022). The ratio shows particularly strong performance for early-onset FGR. A prospective cohort study found an AUC of 0.98 for sFlt-1/PlGF in detecting FGR requiring delivery before 32 weeks, with sensitivity of 100% when the ratio exceeded the 95th centile at 24–28 weeks in a risk-stratified protocol (Herraiz et al., 2018). For risk stratification in suspected preterm FGR, a recent blinded cohort study demonstrated that a positive angiogenic biomarker result, defined as an sFlt-1/PlGF ratio above 38 or PlGF below 100 pg/mL, was associated with a 5-fold higher risk of preterm birth compared with meeting New Zealand FGR criteria alone, whereas the ratio showed limited discrimination in late-onset FGR presenting between 32 and 37 weeks (Hughes et al., 2025). That cohort further confirmed that elevated sFlt-1/PlGF in FGR is independently associated with intrauterine fetal death and adverse neonatal outcomes, supporting its inclusion in routine surveillance protocols. This may explain why clinical trajectories in FGR can deteriorate rapidly once the ratio crosses critical thresholds, even when fetal biometry appears relatively stable (Arnouts et al., 2025).

Circulating cell-free DNA derived from apoptotic syncytiotrophoblast is elevated in FGR and reflects the degree of placental damage and syncytial stress. Exosomal miRNA panels, including miR-210, miR-21, and miR-155, in maternal plasma show altered expression profiles in FGR as early as the second trimester, providing non-invasive windows into placental molecular status (Miranda et al., 2018).

Multi-omics approaches are rapidly emerging as powerful discovery platforms for FGR biomarkers. A landmark systematic review identified 825 non-duplicated metabolites significantly altered in FGR/SGA across 48 studies using 10 different biological sample types, with consistent findings across studies for 56 metabolites including amino acid metabolism pathways and biosynthesis of unsaturated fatty acids in umbilical cord blood (Youssef et al., 2021). Paired maternal-fetal metabolomic profiling has revealed distinct metabolic fingerprints distinguishing FGR from preeclampsia, informing differential diagnosis (Youssef et al., 2021). Machine learning models integrating multi-platform metabolomics with clinical variables have achieved high predictive accuracy for FGR diagnosis in cord blood, with support vector machine algorithms outperforming single-platform approaches (Zimmerman et al., 2025). That review further established that integrating transcriptomics, proteomics, and metabolomics with ML algorithms provides the most comprehensive approach to FGR biomarker discovery, pending validation in prospective cohorts (Rahnavard et al., 2024).

First-trimester combined screening incorporating PAPP-A, PlGF, uterine artery Doppler, mean arterial pressure, and maternal characteristics achieves detection rates of 75%–89% for early-onset FGR requiring delivery before 34 weeks at a 10% screen-positive rate, enabling risk stratification for aspirin prophylaxis (Rolnik et al., 2017). The principal diagnostic biomarkers and surveillance tools currently used in FGR evaluation are summarized in Table 2.

**TABLE 2:** Diagnostic biomarkers of fetal growth restriction.

Category	Biomarker	Clinical utility	Performance/Threshold	References
Biometry	Abdominal circumference (AC)	Most sensitive single parameter; detects hepatic glycogen depletion and fat loss	AC <10th centile (EFW-adjusted), serial velocity assessment	Lees et al. (2020), Papageorghiou et al. (2018)
Biometry	Estimated fetal weight (EFW)	Hadlock formula (HC + AC + FL), INTERGROWTH-21st or WHO charts preferred	EFW <3rd centile = severe FGR, <10th + Doppler abnormality = FGR [1]	Papageorghiou et al. (2018)
Doppler velocimetry	Umbilical artery (UA) PI	Reflects placental vascular resistance, guide for delivery timing	Absent/reversed EDF: emergency delivery indicator	Lees et al. (2020)
Doppler velocimetry	Middle cerebral artery (MCA) PI	Brain-sparing response; cerebroplacental ratio (CPR)	CPR <5th centile: adverse outcome predictor	Lees et al. (2020)
Doppler velocimetry	Ductus venosus (DV) PIV	Late decompensation marker, TRUFFLE trial endpoint	DV PI > 95th centile triggers delivery in TRUFFLE protocol	Lees et al. (2013)
Angiogenic factors	sFlt-1/PlGF ratio	Most validated biochemical marker; short-term prediction of deterioration	Ratio >33: AUC 0.84 (Sen 0.63, Spe 0.84); ratio ≥85: high-risk	Chen et al. (2022), Stepan et al. (2023)
Angiogenic factors	PlGF alone	Low PlGF distinguishes placental FGR from constitutional SGA	PlGF <100 pg/mL at 19–24 weeks: 5× increased FGR risk	Gaccioli et al. (2023)
First-trimester screening	PAPP-A, PlGF and UtA Doppler	Combined screen for early-onset FGR, guides aspirin prophylaxis	Detection rate 75%–89% for early FGR at 10% FPR	Rolnik et al. (2017)
Molecular/omics	Metabolomics panel	825 metabolite alterations identified in FGR/SGA: systematic review	Multi-metabolite ML models: AUC up to 0.91 for FGR prediction	Youssef et al. (2021), Zimmerman et al. (2025)

## 6 Clinical management

**FIGURE 3:**
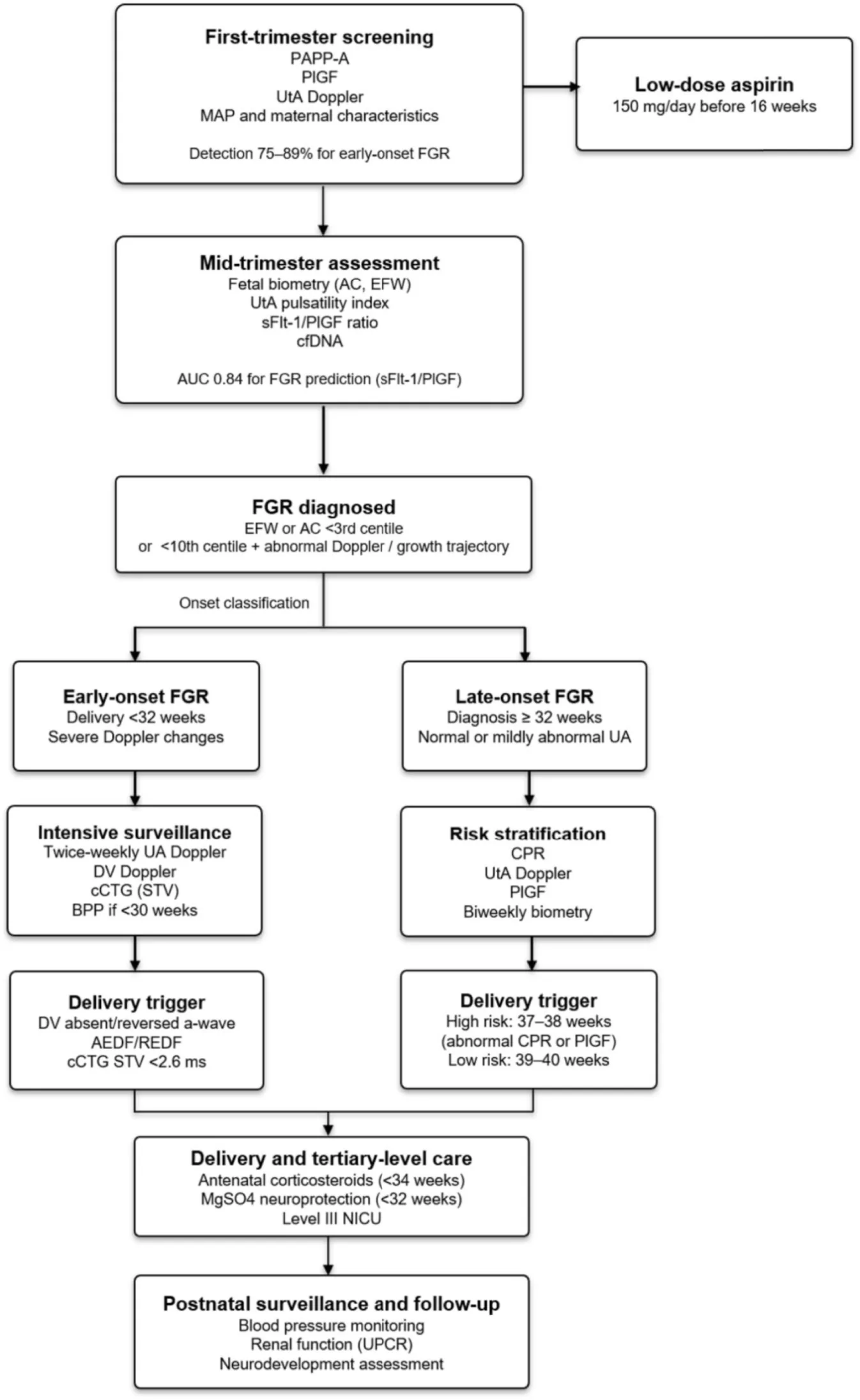
Clinical Screening, Diagnosis and Management Algorithm for Fetal Growth Restriction. A stepwise clinical framework for the identification and management of fetal growth restriction. First-trimester screening integrates maternal factors, PAPP-A, PlGF, mean arterial pressure, and uterine artery Doppler to identify pregnancies at risk and guide aspirin prophylaxis. Mid-trimester assessment combines fetal biometry with angiogenic biomarkers such as the sFlt-1/PlGF ratio. Once FGR is diagnosed, cases are stratified into early-onset and late-onset phenotypes based on gestational age and Doppler findings. Surveillance intensity and delivery timing are subsequently determined using Doppler parameters including umbilical artery, ductus venosus, and cerebroplacental ratio. Postnatal follow-up focuses on cardiovascular, renal, and neurodevelopmental outcomes.

The clinical management of fetal growth restriction is typically guided by a stage-based framework that integrates early pregnancy screening, Doppler-based surveillance, and risk-adapted delivery timing strategies (Figure 3).

### 6.1 Surveillance protocols

Despite broad agreement on the principle that surveillance intensity should escalate with disease severity, how this is operationalized varies substantially across institutions and countries, and this inconsistency has real consequences for clinical outcomes. An international survey revealed that the frequency of assessment, the role of cerebroplacental ratio in guiding management decisions, and the threshold for hospital admission diverged markedly between institutions and countries (Fantasia et al., 2022). A fundamental source of this divergence is the methodological difference between the ISUOG and SMFM frameworks, which weight Doppler parameters, biometric thresholds, and growth trajectory differently, such that the same fetus may receive differing surveillance intensities depending on which guideline a center follows (Lees et al., 2022). The 2023 SOGC guideline sought to bridge this gap through a tiered pragmatic approach, yet acknowledged that the optimal monitoring interval for late-onset FGR with preserved umbilical artery flow remains insufficiently supported by randomized evidence (Kingdom et al., 2023).

### 6.2 Delivery timing

The trials that established current delivery timing frameworks for early- and late-onset FGR were conducted in carefully selected populations, and their generalizability to the heterogeneous clinical populations encountered in routine practice is limited in ways that are not always acknowledged in guideline documents. Post-hoc analyses of the TRUFFLE data revealed that only approximately one-third of infants were delivered according to the pre-specified monitoring criterion, with the majority triggered by safety-net or maternal indications, underscoring the gap between protocol-driven and real-world decision-making (Visser et al., 2017). For late-onset FGR, growing evidence suggests that the DIGITAT framework, which did not differentiate between constitutionally small fetuses and those with true placental insufficiency, may have obscured heterogeneity in treatment effect, a subsequent observational study found that risk-stratified selective induction based on cerebroplacental ratio and uterine artery Doppler yielded meaningfully lower rates of composite adverse neonatal outcome compared with systematic induction at a fixed gestational age (Peasley et al., 2023). The ongoing TRUFFLE-2 and DRIGITAT trials are designed to address precisely these residual uncertainties in monitoring-guided delivery for late preterm and term FGR, and their results are expected to refine the evidence base substantially (Mylrea-Foley et al., 2022).

### 6.3 Prevention and intervention

Low-dose aspirin at 150 mg per day, initiated before 16 weeks of gestation, is currently the most robustly supported pharmacological intervention for preventing early-onset FGR and preeclampsia. The ASPRE trial, a landmark multicenter randomized controlled trial by Rolnik et al. published in the New England Journal of Medicine in 2017, enrolled 1,776 high-risk women identified by first-trimester combined screening and demonstrated a 62% reduction in preterm preeclampsia, defined as delivery before 37 weeks, with corresponding reductions in preterm FGR (Rolnik et al., 2017). Aspirin’s protective mechanisms include improvement of prostacyclin/thromboxane A2 balance, reduction of sFlt-1 release, and improvement of uteroplacental perfusion. This mechanistic profile may explain why aspirin shows its greatest effect when initiated early, before spiral artery remodeling is complete, and has little impact once placental insufficiency is established (Roberge et al., 2018).

Low-molecular-weight heparin (LMWH) has been evaluated as a preventive strategy in women with thrombophilia or recurrent FGR with prior placental abruption or fetal loss. While observational data suggested benefit, the AFFIRM trial found no significant reduction in recurrent FGR with LMWH in an unselected high-risk population, highlighting the need for better patient stratification (Rodger et al., 2016). Sildenafil citrate, a phosphodiesterase-5 inhibitor, was evaluated in the multicenter STRIDER trial. The Netherlands arm was terminated early due to excess neonatal pulmonary hypertension in the treatment group, a result attributed to transplacental transfer and fetal pulmonary vasodilation (Sharp et al., 2018). In contrast, tadalafil, a phosphodiesterase-5 inhibitor with reduced transplacental transfer compared with sildenafil, may reduce fetal and infant deaths, based on the TADAFER-II phase II trial signal, though birth weight improvement was not significant (Pels et al., 2020). These contrasting results underscore the importance of placental pharmacokinetics in FGR drug development.

Nutritional interventions including l-arginine supplementation, which supports nitric oxide synthesis and uterine vasodilation, omega-3 fatty acid supplementation, which promotes uteroplacental angiogenesis, and maternal nutrition optimization remain areas of ongoing research. Melatonin and NAC, by virtue of their antioxidant properties and mitochondrial protective effects, are under investigation as adjunctive therapies to reduce placental oxidative stress in established FGR (Nguyen et al., 2009).

## 7 Neonatal and long-term outcomes

FGR is associated with substantially elevated perinatal morbidity and mortality. Stillbirth risk is increased 4- to 10-fold, accounting for 30%–40% of all stillbirths worldwide (Lawn et al., 2016). In neonates, FGR is associated with respiratory distress syndrome (RDS), necrotizing enterocolitis (NEC), hypoglycemia, polycythemia, and hypothermia, with risk and severity increasing with gestational age at delivery and degree of fetal compromise (Figueras and Gratacos, 2014). Very preterm FGR infants born before 28 weeks of gestation face compounded risks of intraventricular hemorrhage, periventricular leukomalacia, retinopathy of prematurity, and bronchopulmonary dysplasia.

Neurodevelopmental impairment constitutes a major long-term burden. FGR independently confers risk for cerebral palsy, attention deficit hyperactivity disorder (ADHD), executive function deficits, and reduced cognitive performance across childhood and adolescence. A prospective cohort study documented that children born with FGR had significantly lower cognitive scores, higher rates of behavioral problems, and smaller cortical volumes at age 6 years compared with appropriate-for-gestational-age (AGA) controls, findings that persisted after adjustment for gestational age at delivery (Benítez Marín et al., 2022). The underlying mechanisms encompass intrauterine hypoxia-induced neuronal injury, disrupted cortical connectivity, and altered hypothalamic-pituitary-adrenal axis programming.

Organ-level sequelae of FGR are detectable well before adulthood, opening a window for earlier targeted intervention. Evidence of subclinical renal impairment is present from the neonatal period itself: FGR-small-for-gestational-age neonates show significantly elevated urinary protein-to-creatinine ratios and higher mean blood pressure within the first weeks of life compared with appropriately grown preterm peers, consistent with glomerular hyperfiltration secondary to congenital nephron deficit (Sehgal et al., 2025). Structural renal changes persist into adolescence, with MRI studies in very preterm FGR teenagers demonstrating smaller cortical and medullary kidney volumes relative to term-born controls, although filtration markers at that age remain within normal range, suggesting a shrinking reserve rather than overt dysfunction (Liefke et al., 2023). These findings collectively support structured postnatal surveillance of FGR offspring, including serial blood pressure monitoring and renal function assessment, beginning in early childhood rather than waiting for clinical disease to emerge in adult life.

Catch-up growth following FGR is associated with better neurodevelopmental outcomes but paradoxically amplifies cardiometabolic risk when it is rapid and accompanied by adiposity gain. This tension reflects the DOHaD concept of metabolic mismatch and underscores the importance of calibrated postnatal nutritional guidance rather than unrestricted catch-up feeding for FGR infants (Barker, 2004). Published consensus guidelines address the management of SGA/FGR children, including criteria for growth hormone therapy in those with persistent short stature beyond age 4 years (Hokken-Koelega et al., 2023).

## 8 Future perspectives

### 8.1 Single-cell and spatial transcriptomics

The translational impact of single-cell studies in FGR is substantially limited by the systematic underrepresentation of syncytiotrophoblast in standard scRNA-seq workflows. Because the multinucleated syncytiotrophoblast layer is incompatible with microfluidic-based single-cell capture, published placental atlases have consistently recovered STB at far lower proportions than its true biological abundance, and markers conventionally assigned to STB in these datasets likely reflect cytotrophoblast precursors at early fusion states rather than terminally differentiated syncytium (Tang et al., 2024). Single-nucleus RNA sequencing, which isolates nuclei rather than intact cells, partially addresses this limitation but sacrifices cytoplasmic transcripts and further complicates cross-study integration due to systematic batch effects arising from tissue dissociation protocols, gestational age at sampling, and mode of delivery (Elkin et al., 2023). Spatial transcriptomics partially circumvents dissociation bias by retaining positional context, but current commercial platforms resolve transcriptomes at multicellular spot resolution rather than true single-cell resolution, meaning that cell-type deconvolution remains a computational inference rather than a direct measurement. Resolving these technical gaps through cross-platform harmonization and larger FGR-specific cohorts is a prerequisite before these atlases can reliably inform mechanistic hypotheses. In our view, the more immediate priority is not generating additional atlases but developing computational frameworks that can integrate existing datasets while accounting for the systematic biases introduced by differing dissociation protocols and gestational age at sampling.

### 8.2 Trophoblast organoids and precision models

Trophoblast organoid systems have substantially advanced the study of early placentation, but their ability to recapitulate FGR-specific pathology is constrained by several structural limitations. Current villous organoid protocols lack resident immune populations including Hofbauer cells and decidual NK cells, precluding modeling of the immune-trophoblast crosstalk that is central to FGR pathogenesis, separately, the absence of fetal endothelial networks within these organoids means that villous vasculogenesis and the hemodynamic adaptations that deteriorate in FGR cannot be recapitulated *in vitro*. Efforts to incorporate endothelial and immune components through co-culture or assembloid approaches are advancing but have not yet been standardized across laboratories (Haider et al., 2018). Placenta-on-a-chip systems address the hemodynamic dimension by introducing controlled flow and oxygen gradients, but the trophoblast monolayers used in these devices do not replicate the three-dimensional villous architecture of the human term placenta, limiting their capacity to model the structural remodeling defects characteristic of FGR. Bridging these gaps likely requires integration of organoid-derived trophoblast with bioprinted villous scaffolds and patient-derived immune cells to approximate the full complexity of placental dysfunction.

### 8.3 Multi-omics integration and machine learning

The field of multi-omics biomarker discovery in FGR has generated an impressive volume of candidate signatures. Whether any of them will prove reproducible enough to enter clinical practice is a different question, and the answer so far has been discouraging. The central problem is not the absence of candidates but the absence of methodological conditions under which those candidates hold up across independent populations. Virtually all published multi-omics studies in FGR have been conducted in retrospective, single-center cohorts with modest sample sizes, where model performance is evaluated on internal hold-out sets rather than independent external populations, this design systematically overestimates predictive accuracy and cannot account for the platform-specific batch effects that arise when proteomic or metabolomic data are generated across different laboratories, instruments, or sample preservation protocols (Conde-Agudelo et al., 2024). A compounding problem is the timing of sample collection, because FGR is often diagnosed after placental dysfunction is already advanced, most omics datasets reflect the molecular state at a single late-gestational time point rather than the longitudinal trajectory of dysfunction, thereby limiting the ability to distinguish causal from consequential molecular changes and to build models capable of early prediction (Rahnavard et al., 2024). Closing this methodological gap is a prerequisite for multi-omics to move from biomarker discovery to clinical utility. What is needed is not more retrospective discovery cohorts but prospective longitudinal studies with standardized biobanking, pre-registered analysis plans, and external validation across ethnically and geographically diverse population design standards that are routine in cardiovascular biomarker research but have not yet been consistently applied in obstetric multi-omics.

### 8.4 Placental molecular subtyping and targeted therapy

Recognition of the mechanistic heterogeneity of FGR has encouraged the development of precision obstetric strategies in which therapeutic interventions are tailored to the underlying molecular subtype. In some pregnancies, FGR appears to be driven predominantly by oxidative stress and angiogenic imbalance, whereas in others immune dysregulation and epigenetic alterations may play a more prominent role. For the angiogenic subtype, experimental approaches such as recombinant VEGF administration and anti-sFlt-1 RNA interference have demonstrated promising results in preclinical studies. In parallel, immune-targeted strategies are being explored. These include low-dose interleukin-2 therapy aimed at expanding Tregs and interventions designed to promote macrophage polarization toward the M2 or decidual perivascular macrophage phenotype. These approaches remain at an early stage, and whether molecular subtyping will prove sufficiently reproducible to guide individual clinical decisions is, in our view, one of the more important unresolved questions in translational FGR research (Bezemer et al., 2024; Vondra et al., 2023). Whether precision subtyping will translate into meaningfully different outcomes remains to be established. It is unlikely that a single molecular profile will cleanly separate FGR subtypes in real clinical populations, given the degree of pathway overlap documented even within individual placentas. Nanoparticle-mediated placenta-targeted drug delivery systems capable of concentrating therapeutic agents at the trophoblast while minimizing systemic fetal exposure represent a more tractable near-term frontier, with proof-of-concept demonstrations in murine FGR models (Pels et al., 2020). Extracellular vesicle-based therapies that utilize mesenchymal stem cell derived vesicles to deliver anti-inflammatory and pro angiogenic cargo to the placenta are currently under preclinical investigation. Whether any of these strategies will translate into improved neonatal outcomes remains to be established in prospective trials, but their convergence around a common aim, that of matching the therapeutic approach to the biological subtype of FGR, represents a meaningful conceptual shift in how this condition is approached (Tang et al., 2024; Ounadjela et al., 2024).

### 8.5 Translational priorities and research horizons

Not all emerging directions in FGR research are equally close to clinical implementation. Near-term opportunities are likely to come from refinement of angiogenic biomarkers, integration of multi-omics signatures into existing screening frameworks, and machine learning-assisted risk prediction. These approaches build upon established diagnostic pathways and are already being evaluated in prospective cohorts. Molecular subtyping is also attracting increasing attention, although its clinical value will ultimately depend on whether proposed subtypes can be reproduced across diverse populations and linked to meaningful differences in outcome or treatment response.

By contrast, single-cell transcriptomics, spatial profiling, trophoblast organoids, and placenta-on-a-chip systems are unlikely to enter routine clinical practice in the foreseeable future. Their primary value lies in advancing our understanding of placental biology and disease heterogeneity. As the field moves beyond single-pathway explanations of FGR, these technologies may help clarify how distinct molecular programs interact across different clinical phenotypes and stages of disease.

The path toward precision obstetrics is likely to be incremental rather than transformative. Progress will depend not only on identifying new biomarkers or therapeutic targets, but also on establishing clinically meaningful classification systems grounded in biological mechanisms. Experimental models and multi-omics platforms should not be viewed as competing priorities. Rather, they represent complementary components of the same translational continuum (Aye et al., 2025).

## 9 Conclusion

Despite decades of research, FGR remains a leading cause of perinatal morbidity, mortality, and long-term cardiometabolic disease. The central insight from recent molecular work is that FGR does not arise from a single defect. It arises from a convergent, self-reinforcing network of placental pathological mechanisms, and this distinction matters because it explains why interventions that work in isolated experimental models so often disappoint in clinical trials and why surveillance alone, however refined, will not substantially reduce the burden of FGR without mechanistically informed prevention. The network has its origin in impaired EVT invasion and spiral artery remodeling, which drives downstream cascades of oxidative stress, angiogenic imbalance, immune dysregulation, nutrient transport failure, and epigenetic reprogramming that collectively compromise fetal growth. The integration of these mechanistic insights into clinical practice has led to important diagnostic advances. In practice, this has translated into the sFlt-1/PlGF ratio, multi-omics biomarker signatures, and machine learning screening models that are beginning to refine surveillance protocols and inform delivery timing decisions. The advent of single-cell transcriptomics, trophoblast organoids, and precision molecular subtyping promises to move FGR management from the current one-size-fits-all surveillance paradigm toward a mechanistically informed, individualized approach. Progress in this area will require not only scientific advances but also a willingness to design trials that match the mechanistic complexity of FGR rather than simplifying it for operational convenience. Ongoing research efforts directed at translating these molecular insights into effective preventive and therapeutic interventions have potential to substantially reduce the global burden of FGR and its long-term sequelae. Future integration of multi-omics diagnostics with mechanistic therapeutic strategies may transform the management of FGR from surveillance-based care to precision obstetrics.
